# Creatine kinase rate constant in the human heart measured with 3D‐localization at 7 tesla

**DOI:** 10.1002/mrm.26357

**Published:** 2016-08-31

**Authors:** William T. Clarke, Matthew D. Robson, Stefan Neubauer, Christopher T. Rodgers

**Affiliations:** ^1^ Oxford Centre for Clinical Magnetic Resonance Research (OCMR)University of Oxford, John Radcliffe HospitalOxfordUnited Kingdom

**Keywords:** saturation transfer, creatine kinase, high‐energy phosphate, energy metabolism, ^31^P magnetic resonance spectroscopy, phosphorus, cardiac, 7 tesla, 7T

## Abstract

**Purpose:**

We present a new Bloch‐Siegert four Angle Saturation Transfer (BOAST) method for measuring the creatine kinase (CK) first‐order effective rate constant k_f_ in human myocardium at 7 tesla (T). BOAST combines a variant of the four‐angle saturation transfer (FAST) method using amplitude‐modulated radiofrequency pulses, phosphorus Bloch‐Siegert $B_{1}^{+}$‐mapping to determine the per‐voxel flip angles, and nonlinear fitting to Bloch simulations for postprocessing.

**Methods:**

Optimal flip angles and repetition time parameters were determined from Monte Carlo simulations. BOAST was validated in the calf muscle of two volunteers at 3T and 7T. The myocardial CK forward rate constant was then measured in 10 volunteers at 7T in 82 min (after ^1^H localization).

**Results:**

BOAST $k_{f}^{\text{CK}}$ values were 0.281 ± 0.002 s^−1^ in the calf and 0.35 ± 0.05 s^−1^ in myocardium. These are consistent with literature values from lower fields. Using a literature values for adenosine triphosphate concentration, we computed CK flux values of 4.55 ± 1.52 mmol kg^−1^ s^−1^. The sensitive volume for BOAST depends on the B_1_ inhomogeneity of the transmit coil.

**Conclusion:**

BOAST enables measurement of the CK rate constant in the human heart at 7T, with spatial localization in three dimensions to 5.6 mL voxels, using a 10‐cm loop coil. Magn Reson Med 78:20–32, 2017. © 2016 The Authors Magnetic Resonance in Medicine published by Wiley Periodicals, Inc. on behalf of International Society for Magnetic Resonance in Medicine. This is an open access article under the terms of the Creative Commons Attribution License, which permits use, distribution and reproduction in any medium, provided the original work is properly cited.

## 1 INTRODUCTION

The creatine kinase (CK) enzyme catalyzed active spatial transport and temporal buffering of adenosine triphosphate (ATP) in cardiomyocytes (the *CK shuttle*) play a critical role in myocardial energy supply 1, 2, 3, 4. Phosphocreatine (PCr) and the terminal (γ‐) phosphate group of ATP undergo two‐way chemical exchange in the CK shuttle in the mitochondria and in the myofibrils.

Both PCr and ATP give prominent signals during cardiac phosphorus magnetic resonance spectroscopy (^31^P‐MRS). Cardiac ^31^P‐MRS is therefore able to measure the pseudo first‐order rate constant of the CK energy shuttle $k_{f}^{\text{CK}}$ in the human myocardium 5. When combined with absolute quantification of the PCr concentration 6, the total flux of ATP through the CK shuttle (*CK flux*) may be calculated 7. The rate and flux of the CK process has been shown to be a more sensitive measure of the severity of heart failure than the more commonly applied PCr/ATP ratio 8.

To date, measurements of $k_{f}^{\text{CK}}$ in the human heart have been made using pulse sequences without localization, with localization in one dimension, or with outer volume suppression (OVS) only (see Supporting Table S1). Measurements therefore arise from large voxels (with volumes of hundreds of mL). This limits the application of measurements to diffuse, or global, myocardial pathologies rather than specific regions of myocardium affected by disease, for example, infarcts or regions of gross hypertrophy in hypertrophic cardiomyopathy.

Using the signal‐to‐noise ratio improvements brought to human cardiac ^31^P‐MRS by 7 tesla (T) MR 9, 10, this work aims to make the first three‐dimensional (3D)–localized measurements of the CK $k_{f}^{\text{CK}}$ in the human myocardium. Three‐dimensional localization will enable measurement of $k_{f}^{\text{CK}}$ from smaller, more sharply delineated voxels and will reduce the dependence of voxel shapes on the receive coil. This means that measurements can be better targeted for applications in localized disease and may make measurements more robust against contamination by signals from skeletal muscle.

## 2 THEORY

Since 2000, four saturation‐transfer methodologies have been used to measure myocardial $k_{f}^{\text{CK}}$ and CK flux in the human heart:

- The four‐angle saturation transfer (FAST) method, developed for $k_{f}^{\text{CK}}$ measurement at 1.5T 11.
- Triple repetition time saturation transfer (TRiST), developed for 3T 12.
- Two repetition time saturation transfer (TwiST), developed for 3T (similar to TRiST but with decreased measurement duration) 13.
- Time‐dependent saturation transfer (TDST), implemented at 3T for reproducibility measurements of $k_{f}^{\text{CK}}$ 14.

Without localization, the $k_{f}^{\text{CK}}$ measured would include significant contamination from skeletal muscle, which has different high‐energy phosphate kinetics. The methods listed above have all been acquired using 1D localization schemes (1D‐chemical shift imaging [CSI], 1D‐image selected in vivo spectroscopy), or using OVS, to measure myocardial $k_{f}^{\text{CK}}$ values.

### 2.1 Compatibility of Existing Methods at 7T

Cardiac ^31^P‐MRS is typically performed using surface coils for transmit and receive. The FAST, TRiST, TwiST, and TDST methods all require $B_{1}^{+}$‐insensitive adiabatic pulses (e.g., BIR‐4 or adiabatic half‐passage pulses) to generate a spatially invariant excitation flip angle when transmitting with a surface coil. However, it is challenging to achieve sufficient $B_{1}^{+}$ to surpass the adiabatic onset for such pulses in the heart at 7T. Increasing the pulse duration would not be a solution because it produces undesirably high levels of radiofrequency (RF) heating (specific absorption rate) and T_2_ dephasing of the spin‐locked magnetization during the pulse 15. Therefore, none of the currently available methods are suitable for use at 7T without modification. Supporting Table S1 summarizes the compatibility for use at 7T of CK measurement protocols from the literature 11, 12, 14, 16, 17, 18, 19.

The FAST method does not require specific flip angles; rather, the flip angles must be known accurately, which in the original 1.5T implementation was achieved using BIR‐4 pulses. Therefore, the BIR‐4 pulses may be substituted for amplitude modulated pulses if the flip angle can be accurately calibrated. A Bloch‐Siegert–based $B_{1}^{+}$‐mapping technique for ^31^P‐MRS suitable for this purpose was recently published by the authors 20.

All cardiac 7T ^31^P RF transmit coils in Oxford are of a surface coil design 9, 21, 22; thus, the flip angle across the myocardium will vary significantly. Therefore, even if we measure $B_{1}^{+}$, as described in 20, we must still localize to voxels small enough to experience a narrow intravoxel range of flip angles. This may be achieved by using 3D localization, for example, 3D‐CSI or 2D‐CSI and slice selection. Acquiring 3D‐resolved spectra from comparatively small voxels also minimizes intravoxel B_0_ inhomogeneity, which might otherwise lead to unacceptably broad linewidths at 7T.

Although published implementations of FAST have used 1D‐CSI localization, FAST should be compatible with other localization schemes because it requires no fully relaxed (long repetition time [TR]) scan, whereas TRiST and TwiST do require such a scan and would have prohibitively long total durations for 2D‐ or 3D‐resolved variants (see Supporting Table S1 for details). Therefore, FAST was selected as the basis for our 7T 3D‐localized $k_{f}^{\text{CK}}$ protocol.

### 2.2 Recap of FAST

FAST 11 uses $B_{1}^{+}$‐insensitive BIR‐4 (or BIR‐phase cycled [BIRP] 23) pulses to produce constant flip angles of 15° (denoted by α) and 60° (denoted by β) in spite of inhomogeneous $B_{1}^{+}$ from a surface coil. One‐dimensional CSI data are collected twice for each flip angle: first with saturation of the γ‐ATP peak and then with saturation mirrored on the other side of the PCr peak (control saturation). FAST is effectively two dual‐angle T_1_ measurements: one with γ‐ATP saturation and one without 24. The relaxation time under steady‐state saturation ( $T_{1}^{\prime }$) and under control saturation (written simply as $T_{1}$), may be calculated thus 24:

(1) $$T_{1}^{\left(\prime \right)}=-T_{R}/\mathrm{In}\left[\frac{\sin\;\alpha -R^{\left(\prime \right)}\sin\;\beta }{\cos\;\beta \;\sin\;\alpha -R^{\left(\prime \right)}\cos\;\alpha \;\sin\;\beta }\right],$$

where $R^{\left(\prime \right)}=M^{\left(\prime \right)}\left(\alpha \right)/M^{\left(\prime \right)}\left(\beta \right)$ is the ratio of the partially saturated PCr amplitudes with the two flip angles α and β, and (‘) means Equation [1] holds both with and without saturation. Then, Equation [1] and the partial saturation equation:

(2) M(θ)=sin(θ)M0(1−e−TRT1)1−cos θ e−TRT1,

are combined to calculate $M_{0}^{\left(\prime \right)}$ values from either of the PCr amplitudes, $M^{\left(\prime \right)}\left(\alpha \right)\;\;\text{or}\;M^{\left(\prime \right)}\left(\beta \right)$, recorded under the same saturation conditions:

(3) $$\begin{array}{c} M_{0}^{\left(\prime \right)}=\frac{M^{\left(\prime \right)}\left(\alpha \right)\left[\cos\;\beta -\cos\;\alpha \right]}{\sin\;\alpha \left[\cos\;\beta -1\right]-R^{\left(\prime \right)}\sin\;\beta \left[\cos\;\alpha -1\right]}\\ =\frac{M^{\left(\prime \right)}\beta \left[\cos\;\alpha -\cos\;\beta \right]}{\sin\;\beta \left[\cos\;\alpha -1\right]-\sin\;\alpha \left[\cos\;\beta -1\right]R^{\left(\prime \right)-1}}. \end{array}$$

Assuming steady‐state saturation of the γ‐ATP peak, the CK rate constant is given by:

(4) $$k_{f}^{\text{CK}}=\frac{1}{T_{1}^{\prime }}\left(1-\frac{M_{0}^{\prime }}{M_{0}}\right).$$

Previous values of $k_{f}^{\text{CK}}$ and CK flux measured by FAST are given in Table 1.

**Table 1:** Summary of Results from Recent Studies Measuring Cardiac $k_{f}^{\text{CK}}$ and CK Flux.

Reference	Method	Field (T)	Localization	Group	N	PCr/ATP	[PCr] mmol kg^−1^	[ATP] mmol kg^−1^	$k_{f}^{\text{CK}}$ s^−1^	CK Flux mmol kg^−1^ s^−1^
Weiss, 2005 7	FAST	1.5	1D CSI	Normal (rest)	16	–	10.1 ± 1.3	5.7 ± 1.3	0.32 ± 0.07	3.2 ± 0.9
				Normal (stress)	6	–	9.9 ± 1.2	5.6 ± 1.4	0.33 ± 0.09	3.3 ± 1.2
				CHF	17	–	8.3 ± 2.6	5.2 ± 1.3	0.21 ± 0.07	1.6 ± 0.6
Smith, 2006 8	FAST	1.5	1D CSI	Normal	14	1.9 ± 0.3	9.4 ± 1.1	5.5 ± 1.3	0.32 ± 0.06	3.1 ± 0.8
				LVH	10	1.3 ± 0.3	6.1 ± 2.0	4.7 ± 1.3	0.36 ± 0.04	2.2 ± 0.7
				LVH + CHF	10	1.3 ± 0.3	7.2 ± 3.7	5.0 ± 1.1	0.17 ± 0.06	1.1 ± 0.4
Bottomley, 2009 34	FAST	1.5	1D CSI	Normal	15	1.9 ± 0.5	9.6 ± 1.1	5.5 ± 1.3	0.33 ± 0.07	3.2 ± 0.8
				MI	15	1.7 ± 0.3	5.4 ± 1.2	3.4 ± 1.1	0.31 ± 0.08	1.7 ± 0.5
Abraham, 2013 35	FAST	1.5	1D CSI	Normal	17	1.7 ± 0.3	9.4 ± 1.2	5.8 ± 1.2	0.38 ± 0.07	3.6 ± 0.9
				HC	9	1.4 ± 0.4	7.8 ± 2.3	5.0 ± 0.8	0.28 ± 0.15	2.0 ± 1.4
Schar, 2010 12	TRiST	3	1D CSI	Normal	8	–	–	–	0.32 ± 0.07	–
Schar, 2015 13	TwiST	3	1D CSI	Normal	12	–	–	–	0.33 ± 0.08	–
				CHF	17	–	–	–	0.20 ± 0.06	–
Bashir, 2014 14	TDST	3	1D ISIS	Normal	15	1.9 ± 0.2	10.5 ± 0.8	5.5	0.32 ± 0.05	3.3 ± 0.6
All^a^				Normal	97	1.9 ± 0.1	9.8 ± 0.4	5.6 ± 0.1	0.33 ± 0.02	3.3 ± 0.2
All^a^				CHF	44	1.3	7.8 ± 0.6	5.1 ± 0.1	0.19 ± 0.07	1.3 ± 0.3
This study's results.										
This study^b^	BOAST	7	3D CSI	Normal	10	1.96 ± 0.44	11.39 ± 2.59	5.5	0.37 ± 0.04	4.45 ± 1.49
This study^c^	BOAST	7	3D CSI	Normal	10	–	–	–	0.35 ± 0.05	–

ATP, adenosine triphosphate; BOAST, Bloch‐Siegert four angle saturation transfer; CHF, chronic heart failure; CK, creatine kinase; CSI, chemical shift imaging; FAST, four‐angle saturation transfer; HC, hypertrophic cardiomyopathy; ISIS, image selected in vivo spectroscopy; LVH, left ventricular hypertrophy; MI, postmyocardial infarction; N, number; PCr, phosphocreatine; T, telsa; TDST, time‐dependent saturation transfer; TRiST, triple repetition time saturation transfer; TwiST, two repetition time saturation transfer.

a Weighted arithmetic means of the normal and chronic heart failure (CHF) groups.

b Using least‐squares fitting.

c Using equations [(1), (2), (3), (4)].

### 2.3 The Bloch‐Siegert Four Angle Saturation Transfer Method

**Figure 1:**
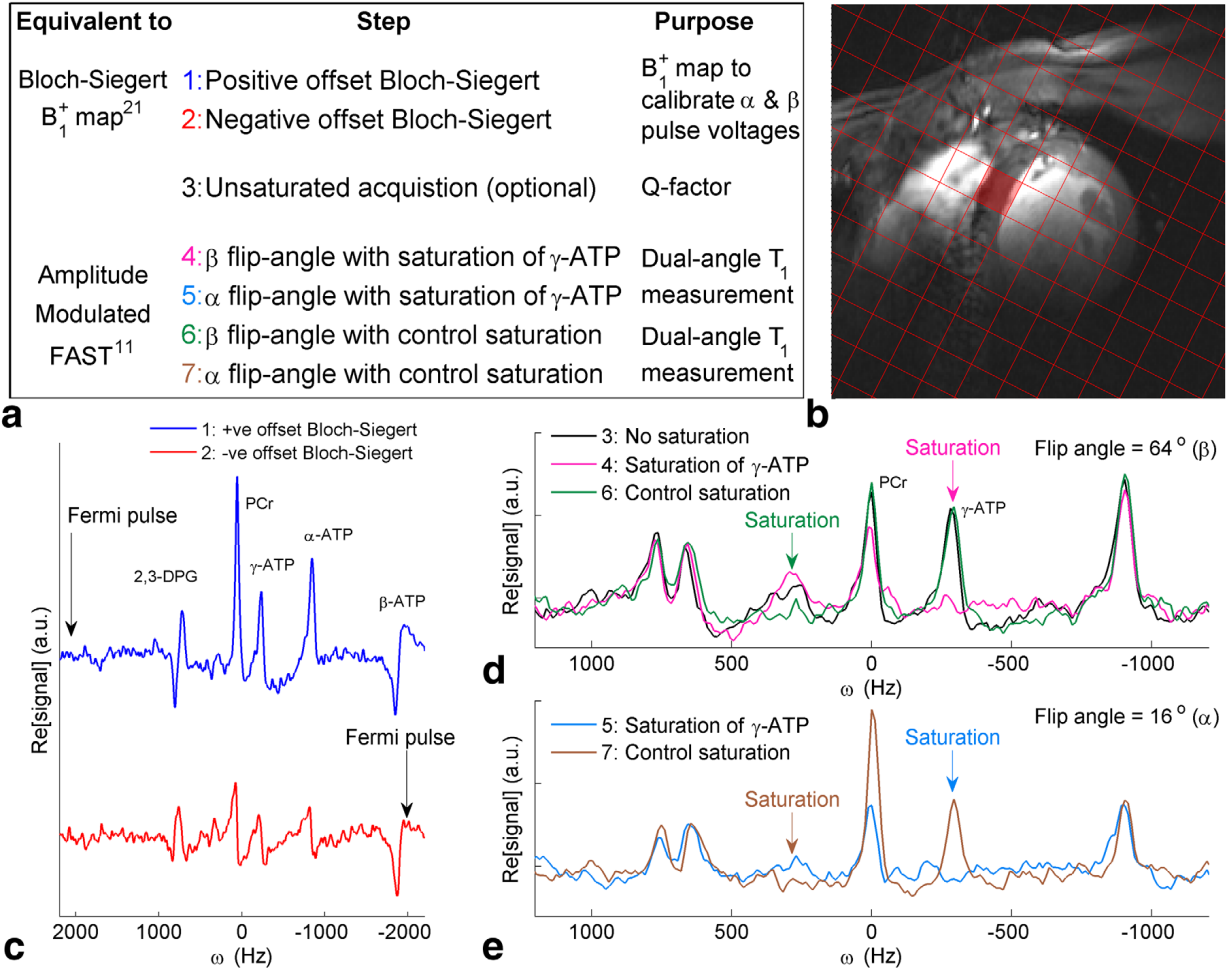
**(a)** Summary of Bloch‐Siegert four‐angle saturation transfer protocol steps. **(b)** ^1^H mid‐short‐axis localizer with the chemical shift imaging matrix overlaid and the target septal voxel shaded. (c‐e) Spectra from that target voxel. **(c)** Bloch‐Siegert $B_{1}^{+}$ mapping scans (steps 1‐2). **(d)** Unsaturated, control saturation, and saturated scans at FA = β. **(e)** Control and saturated scans at FA = α. Note that the PCr signal‐to‐noise ratio in scan 6 (FA = β, control saturation scan) was 17.5. ATP, adenosine triphosphate; FA = flip angle; FAST, four‐angle saturation transfer; PCr, phosphocreatine.

We propose the following Bloch‐Siegert four Angle Saturation Transfer (BOAST) protocol to measure 3D‐resolved $k_{f}^{\text{CK}}$ in the heart at 7T (Fig. 1a):

Acquire a 3D‐resolved ^31^P $B_{1}^{+}$ map:

1. Positive‐offset Bloch‐Siegert acquisition;
2. Negative‐offset Bloch‐Siegert acquisition;
  Use the $B_{1}^{+}$ map to calculate transmit voltages to deliver desired α and β flip angles at the target voxel(s), then:
3. Optionally acquire a nonsaturated CSI data set at α or β flip angle for frequency calibration and measurement of direct saturation;
4. Acquire at β flip angle with γ‐ATP saturation;
5. Acquire at α flip angle with γ‐ATP saturation;
6. Acquire at β flip angle with control saturation;
7. Acquire at α flip angle with control saturation;

Finally, fit spectra in each voxel using the advanced method for accurate, robust, and efficient spectral fitting (AMARES) 25, 26 and calculate $k_{f}^{\text{CK}}$ using Equations [(1), (2), (3), (4)].

This protocol enables $k_{f}^{\text{CK}}$ to be measured across a 3D‐CSI matrix (Fig. 1b) without requiring the use of adiabatic pulses. However, as described below, the accuracy and precision of the measurement in each voxel will depend on the flip angles achieved there. These flip angles may be chosen freely for one target voxel, but they will vary elsewhere according to the coil transmit profile. Note, however, that the ratio of flip angles m = β/α (assuming without loss of generality that m > 1) is fixed over all space by the ratio of transmit voltages (m = V_β_/V_α_) chosen by the operator.

### 2.4 Minimum Saturation Time for Steady‐State Saturation

Equation [4] strictly only holds for experiments that maintain a saturated steady state, that is, selective saturation of γ‐ATP is continuous throughout the experiment so that the magnetization $M^{\gamma -\text{ATP}}=0$ at all times.

Delay alternating with nutation for tailored excitation (DANTE) pulse trains 12, 27 are used to create continuous selective saturation. Bloch simulations and phantom tests (not shown) established that a fixed amplitude DANTE train ( $m=1,T_{\text{on}}=100\;\mu s,\;T_{\text{off}}=230\;\mu s,$ in the notation of reference 12) provided saturation across the range of $B_{1}^{+}$ values expected in the interventricular myocardium (50–800 Hz $\gamma B_{1}^{+}$) while maintaining a Q (the ratio of PCr M_0_ measured with control saturation to that measured with no saturation 11, ideally 1.00) of > 0.95 at a 289 Hz (2.4 ppm) frequency offset, that is, the PCr‐ to γ‐ATP separation at 7T.

Full‐sequence Bloch simulations (not shown) indicate that the saturation time should exceed 76% of the T_R_. Steps 3 to 7 of BOAST will be run with 512 spectral points and a 6 kHz bandwidth, that is, with 85 ms readouts. Additional sequence components (excitation, spoiling, etc.) increase the time without saturation to 90 ms. Therefore, the minimum T_R_ is 375 ms, which we rounded to 400 ms.

## METHODS

### 1 Effect of Flip Angle and Repetition Times for BOAST

Monte‐Carlo Bloch simulations of the pair of scans in the BOAST protocol with and without γ‐ATP saturation were performed to explore how they affect the accuracy and precision of BOAST $k_{f}^{\text{CK}}$ values. Bloch simulations were performed ignoring localization for two exchanging peaks at a range of repetition times (0.3–2.0 s) and flip angles (1 °–180 °), with control‐ and on‐resonance (γ‐ATP) saturation. Other parameters were matched to the best known values for PCr and γ‐ATP in the heart ([PCr]:[ATP] = 1.5:1, $T_{1}^{*,PC_{r}}=6.0\;s,\;T_{1}^{*,\gamma -\text{ATP}}=2.0\;s,\;k_{f}^{\text{CK}}=0.32\;s^{-1}$) 28. The resultant magnetization was then used to generate a distribution of values (50,000 points) at each combination of input variables. The standard deviation (SD) of the distribution was scaled to be equal to the Cramér–Rao lower bound (CRLB) of cardiac ^31^P MR spectra, which was acquired with the hardware that we planned to employ for BOAST scans and scaled for the variable $T_{R}\left(\text{CRLB}/\sqrt{T_{R}}\right)$ 9. The simulation was then repeated with Monte Carlo SDs equal to 0.75, 1.25, and 1.5 × the CRLB. The values of $T_{1}^{{\text{PC}}_{r}},M_{0}^{{\text{PC}}_{r}}$, and $k_{f}^{\text{CK}}$ were separately calculated for each repetition in each distribution, and means and SDs were calculated.

### 2 Bloch‐Siegert CSI Resolution

To determine the optimal Bloch‐Siegert $B_{1}^{+}$ map resolution, Bloch‐Siegert $B_{1}^{+}$ maps were acquired at a range of resolutions 8, 12, 16, 20, 26, 32 in the axis perpendicular to the coil, from a uniform phantom (120 × 270 × 270 mm^3^ box containing 40mM K_2_HPO_4(aq)_). The field of view (FOV) was 200 × 330 × 330 mm^3^; the other two dimensions were fixed at a resolution of 16; and k‐space was acquisition weighted.

The maps collected with high resolution in the first dimension (CSI matrix > 20, nominal voxel size < 10 mm) were zero‐padded in k‐space to 256 × 256 voxels and averaged to form a high‐resolution true $B_{1}^{+}$ profile. The interpolated maps were used to simulate the signal arising from a FAST experiment (selectively saturated and unsaturated steady‐state CSI spectra of PCr and γ‐ATP in exchange) at α and β flip angles scaled to the true $B_{1}^{+}$ at each of the high‐resolution points. The point spread function (PSF) and receive weighted (approximated as the normalized $B_{1}^{+}$ profile) simulated FAST signal were then summed for each voxel for each of the original resolution grids. A $k_{f}^{\text{CK}}$ value was then calculated from the summed signal using the original $B_{1}^{+}$ measured at each resolution. The error in the final $k_{f}^{\text{CK}}$ was expressed as a percentage error on color maps overlaying ^1^H localizers of the phantom.

### 3 Experimental Methods

Unless stated otherwise, the experiments below used a Magnetom 7T scanner (Siemens, Erlangen, Germany). Localizer images were acquired with a 10‐cm ^1^H loop coil (Rapid Biomedical, Rimpar, Germany), which was replaced by a transmit/receive (T/R) switch and preamplifier module (Virtumed LLC, MN), connected to a 10‐cm ^31^P T/R loop coil for the ^31^P acquisitions, and the ^31^P coil was tuned and matched using an RF sweeper (Morris Instruments Inc., Ottawa, Canada) for each subject. All subjects were recruited in a manner approved by the local research ethics committee.

### Skeletal Muscle Validation at 3 Tesla

BOAST was validated against literature $k_{f}^{\text{CK}}$ values in skeletal muscle in the human calf, as follows.

#### 1 Tesla Volume Transmit/Receive Coil

The calf of one volunteer (male, 23 years, 84 kg) was scanned using a ^1^H/^31^P dual‐tuned birdcage head coil (Rapid Biomedical, Rimpar, Germany) in a 3T Trio scanner (Siemens, Erlangen, Germany). The volume coil has a comparatively uniform excitation field, which enables comparison of the BOAST method with previous implementations of FAST using BIR‐4 pulses. Using 3T (not 7T) and a volume receive coil (not a surface receive coil) will result in lower SNR and a less precise $k_{f}^{\text{CK}}$ measurement than we ultimately anticipate at 7T.

BOAST scanning was performed following the protocol above. Bloch‐Siegert scans were performed with a 12 × 12 × 8 CSI matrix; 150 × 180 × 300 mm^3^ field of view; 1,024 spectral points; 4 kHz bandwidth centered at PCr; 70 V_RMS_ (at the coil plug)‐shaped excitation pulse^9^; $T_{R}=0.5\;s$; and 15 averages at k = 0—giving a total duration for each scan of 10 min. The Bloch‐Siegert Fermi pulse parameters were $T_{P}=3.5\;\text{ms}$, $\;T_{0}=0.875\;\text{ms}$, and  a=0.224 ms at 250 V. The Fermi pulse was positioned ±2,000 Hz from PCr. The $B_{1}^{+}$ map was calculated as previously described in 20.

The acquisition parameters for the subsequent saturation transfer steps were as above, except with 512 spectral points. The $T_{R}=0.4\;s$ with 21 averages at the center of k‐space; the duration of each scan was 10 min. Selective saturation was achieved by DANTE pulses ( $m=1,T_{\text{on}}=100\;\mu s,\;T_{\text{off}}=230\;\mu s,\;\text{at}\;50\;V$). The excitation pulse was scaled using the acquired $B_{1}^{+}$ maps to give the optimal flip angles of α=16°,β=64°.

Spectra were fitted using AMARES in MATLAB (MathWorks, Natick, MA) 26 with prior knowledge specifying 10 Lorentzian peaks (α,β,γ‐ATP multiplet components, PCr, PDE, and P_i_) and fixed amplitude ratios and scalar couplings for the multiplets. The voxels inside the leg in the four central slices of the CSI grid (head–foot direction) were analyzed.

$k_{f}^{\text{CK}},T_{1}^{*}$, and $M_{0}^{\text{PCr}}$ were calculated using Equations [(1), (2), (3), (4)]. In addition, the Q was determined as the ratio of the PCr amplitude measured with control saturation to the PCr amplitude measured in an additional acquisition without saturation.

#### 2 Surface Coil (7T)

The experiment was repeated in another volunteer's (male, 24 years, 70 kg) calf at 7T using an identical experimental protocol. The acquisition parameters for the Bloch‐Siegert scans were 12 × 12 × 14 CSI matrix; 180 × 200 × 300 mm^3^ field of view; 2,048 spectral points; 6 kHz bandwidth centered at PCr; the shaped excitation pulse at 250 V; $T_{R}=0.5\;s$; and 7 averages at k = 0, giving a scan duration of 10 min. The Bloch‐Siegert Fermi pulse parameters were as above.

For the subsequent saturation transfer steps, 512 spectral points were used; $T_{R}=0.4\;s$ with 5 averages at the center of k‐space, for a scan duration of 7 min. Selective saturation was achieved by hard DANTE pulses at 33 V. The excitation pulse was scaled using the acquired $B_{1}^{+}$ maps to give flip angles of α=16°,β=64°, at the center of the calf. Spectra from the central five slices of the CSI grid were masked and analyzed as for 3T. An additional mask was created to select voxels within 20% of the target flip angles; these voxels were also analyzed separately.

### 4 Cardiac Validation

Ten healthy subjects (8 male, 2 female, 31 ± 8 years and 71 ± 8 Kg) underwent the cardiac protocol. Subjects were positioned, and localizers were acquired as previously described 9. The experimental protocol followed the steps set out for BOAST above.

The Bloch‐Siegert scans used a 16 × 6 × 8 CSI matrix; 240 × 240 × 200 mm^3^ field of view; 2,048 spectral points; 6 kHz bandwidth centered at PCr with a $T_{R}=0.5\;s$; and 11 averages at k = 0. The duration of each of steps 1 and 2 was 15:05 min. Excitation used the shaped excitation pulse at 270 V. The Bloch‐Siegert Fermi pulse parameters were $T_{P}=0.875\;\text{ms}$, $T_{0}=0.875\;\text{ms}$, a=0.224 ms, at 240 V. The Fermi pulse was positioned at ± 2,000 Hz from PCr.

For the subsequent saturation transfer steps, the spectral points were set to 512; the $T_{R}=0.4\;s$; and the number of averages at the center of k‐space was 11 such that the duration of each scan was 10:22 min. Selective saturation was achieved by DANTE pulses with a transmit voltage of 33 V. The total ^31^P acquisition time was 81:50 min for the complete scan.

A target voxel was selected at scan time from the center of the mid interventricular septum, and the transmit voltage was adjusted to set α=16°,β=64° there. Spectra from the target voxel were fitted using AMARES in MatLab (MathWorks), with prior knowledge specifying 11 Lorentzian peaks (α,β,γ‐ATP multiplet components, PCr, PDE, and 2 × 2,3‐diphosphoglycerate [2,3 DPG]) and fixed amplitude ratios and scalar couplings for the multiplets.

$k_{f}^{\text{CK}},T_{1}^{*}$, and $M_{0}^{\text{PCr}}$ were calculated from the PCr amplitudes using Equations [(1), (2), (3), (4)]. The CRLB of each derived parameter and the Q were also calculated.

#### 4.1 Multiparametric Fitting

In addition to the analysis described above, the amplitudes of the PCr and unsaturated γ‐ATP and α‐ATP peaks were least‐squares fitted using analytical Bloch‐McConnell expressions for two‐pool mixing (given in Appendix A). The variance‐weighted, nonlinear least squares fitting of $M_{0}^{\text{PCr}},M_{0}^{\text{ATP}},T_{1}^{*,PCr},T_{1}^{*,\gamma -\text{ATP}},T_{1}^{\alpha -\text{ATP}}$, and $k_{f}^{\text{CK}}$ was done using lsqcurvefit in MATLAB (MathWorks) 29. This method also enables computation of the unsaturated PCr/ATP ratio $\left(M_{0}^{\text{PCr}}/M_{0}^{\text{ATP}}\right)$.

An average literature value for the concentration of ATP in the myocardium was calculated (Table 2) and used to calculate [PCr]:

(5) $$\left[PCr\right]=\left[ATP\right]\frac{M_{0}^{\text{PCr}}}{M_{0}^{\text{ATP}}}.$$

**Table 2:** Reported PCr and ATP Concentrations.

Reference	[ATP] (mmol kg^−1^)	[PCr] (mmol kg^−1^)
Bottomley, 1990 40	6.90 ± 1.60	11.00 ± 2.70
Yabe, 1995 41	7.72 ± 2.97	12.14 ± 4.25
Bottomley, 1996 6	5.80 ± 1.60	10.00 ± 2.00
Okada, 1998 42	6.40 ± 1.80	9.70 ± 2.50
Meininger, 1999 43	5.30 ± 1.20	9.00 ± 1.20
Beer, 2002 44	5.69 ± 1.02	8.80 ± 1.30
El‐Sharkawy, 2013 15	6.00 ± 1.10	10.40 ± 1.50
Weiss, 2005 7	5.70 ± 1.30	10.10 ± 1.30
Smith, 2006 8	5.50 ± 1.30	9.40 ± 1.10
Bottomley, 2009 34	5.50 ± 1.30	9.60 ± 1.10
Abraham, 2013 35	5.80 ± 1.20	9.40 ± 1.20
Average	5.82 ± 0.40	9.58 ± 0.44

Reported values of [ATP] and [PCr] measured by ^31^P‐MRS in human myocardium. The averages are variance weighted means of the study means.

ATP, adenosine triphosphate; PCr, phosphocreatine.

From the per‐subject PCr concentration, the CK flux was calculated as the product of $k_{f}^{\text{CK}}$ and [PCr].

## 3 RESULTS

### 3.1 Effect of Flip Angles and Repetition Times for BOAST

**Figure 2:**
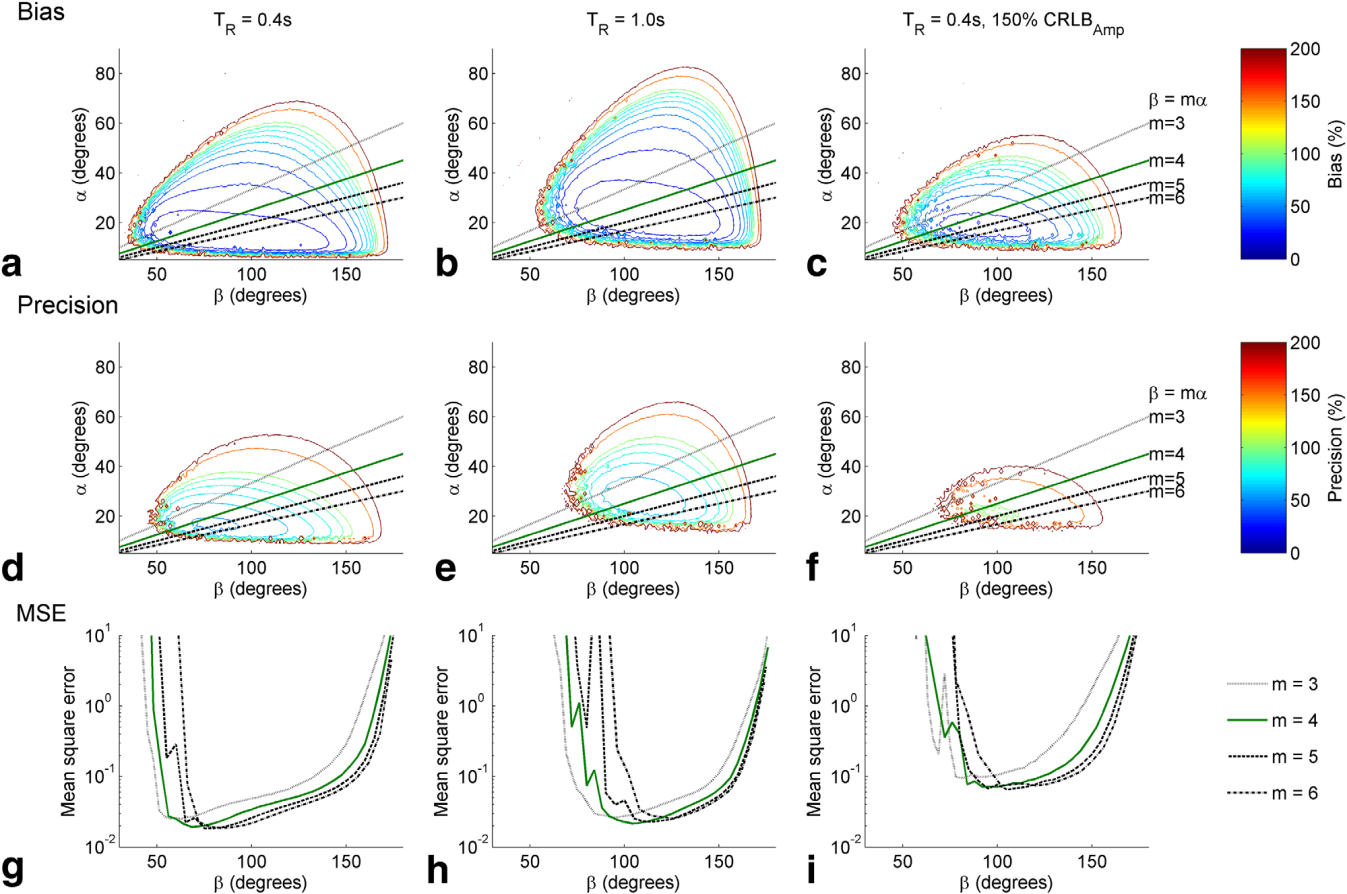
Monte‐Carlo simulations of steps 4‐7 in BOAST (equivalent to a FAST experiment). **(a‐c)** The predicted measurement bias as a function of the two flip‐angles (α and β) at two repetition times and at a lower SNR (50% increase in amplitude Cramér–Rao lower bound). The black lines mark the possible flip angles at different values of *m*. **(d‐f)** The predicted measurement precision, in percent. **(g‐i)** Cross sections of the mean square error, plotted on a logarithmic scale, at the points along the lines in plots **a‐b** and **d‐f** for the listed values of *m*. BOAST, Bloch‐Siegert four angle saturation transfer; FAST, four‐angle saturation transfer; MSE, mean square error; SNR, signal‐to‐noise ratio; T_R_, repetition time.

**Figure 3:**
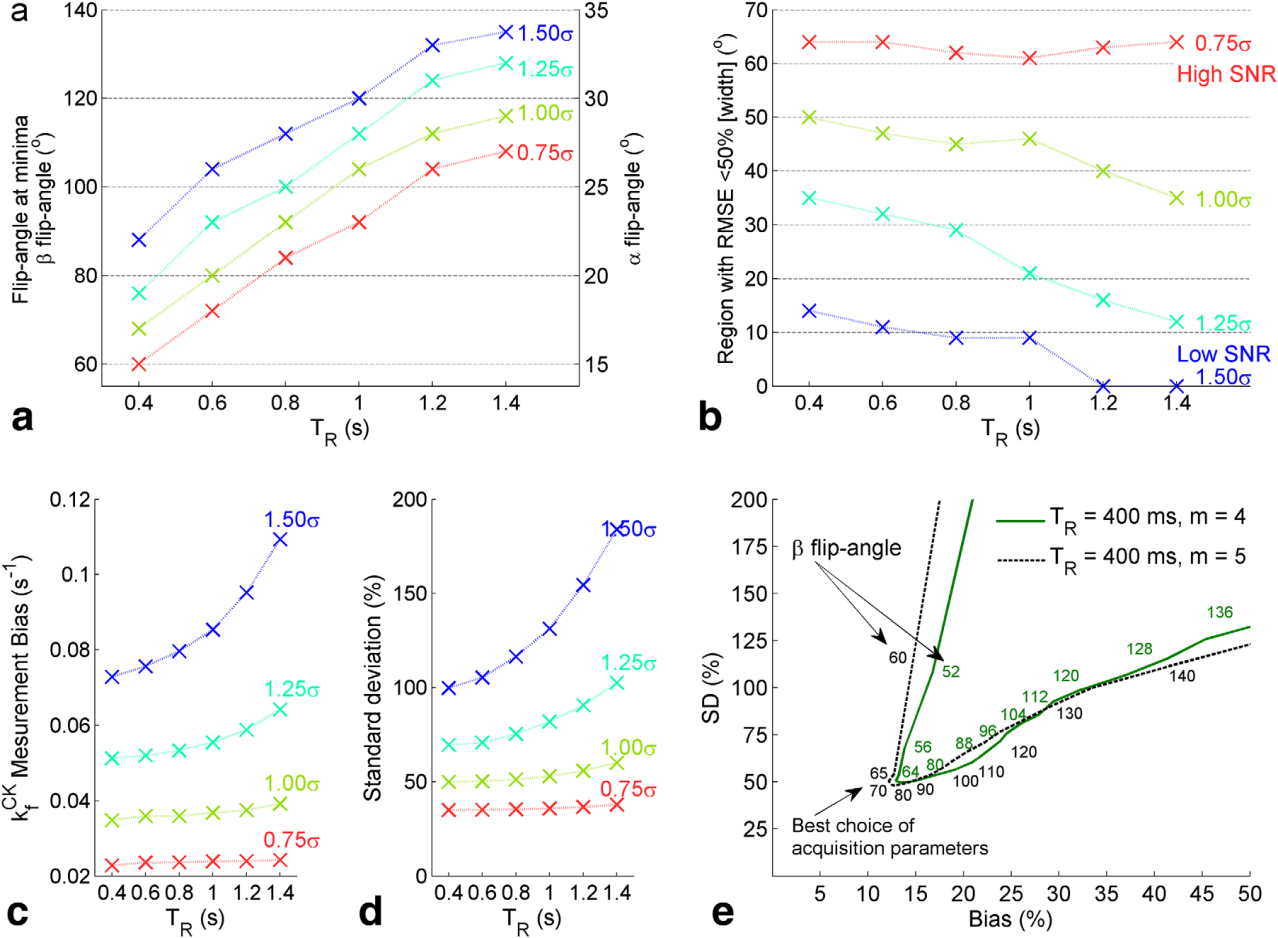
Further analysis of Monte Carlo simulation results shown in Figure 2. **(a)** Position of the mean square error minima, for *m=4*, as a function of repetition time and at different values of PCr amplitude SD (σ). The SD value used for 1.00σ is equal to the amplitude Cramér—Rao lower bound reported in cardiac spectra from the same apparatus 9 **(b)** Width, measured in degrees, of the region with acceptable RMSE (defined as less than 50% of the $k_{f}^{\text{CK}}$). (**c** and **d**) Predicted measurement bias **(c)** and percentage SD (**d**) of the experiment at the minima in Figure 3b. **(e)** L‐curves of the cross‐sections shown in Figure 2c. The axes are percentage bias and precision (SD); the optimum measurement point corresponds to the inflection in each line. The effect of changing *m* is small. CK, creatine kinase; PCr, phosphocreatine; RMSE, root mean square error; SD, standard deviation; SNR, signal‐to‐noise ratio; T_R_ = repetition time.

An example of the simulated data is shown, for a range of $T_{R}$ values and SNR levels, in Figure 2. Analysis of the minimum bias (Fig. 3c) and SD (Fig. 3d), and the width of regions with acceptable mean square error (Fig. 3a), showed that lower T_R_ values gave higher precision and accuracy. L‐curve analysis showed that β/α ratios of 4 and 5 give similar results (Fig. 3e), but a ratio of 4 was chosen because it gives a (slightly) larger acceptable range of $B_{1}^{+}$ values relative to the target. The optimal pair of flip angles was chosen as the point with minimum mean square error. With a minimum T_R_ of 400 ms (see Theory), the optimal protocol parameters were $T_{R}=0.4\;s,\;\;\alpha =16{}^{\circ},\beta =64{}^{\circ}$. This choice of flip angles is close to that recommended by Bottomley and Ouwerkerk for conventional adiabatic FAST 24, although the $T_{R}$ for BOAST is much shorter.

Increasing the Monte Carlo SD, equivalent to lowering the SNR of the system, affects the width and position of the region of flip angles with acceptable bias and precision (Figs. 3a and b). For example, the equivalent protocol run at 3T (a 2.8 times lower SNR) has an acceptable error region 0.13 times the size of the region at 7T, the optimum flip angles at 3T with a T_R_ = 400 ms are β = 112, α = 28. A lower SNR increases the sensitivity the protocol to the choice of parameters (Figs. 3c and d).

Changing the ratio of metabolite concentrations ([PCr]:[ATP]), or the exchange rate ( $k_{f}^{\text{CK}}$), in the simulation by ± 25% results in a less than 5% change in measurement bias and less than 25% change in SD when using the chosen optimal flip angle and repetition time and using an unmodified Monte Carlo SD. A larger change in simulation parameters ( ± 50%) results in a less than 50% change in measurement bias and less than 100% change in SD.

### 3.2 Effect of CSI Resolution

**Figure 4:**
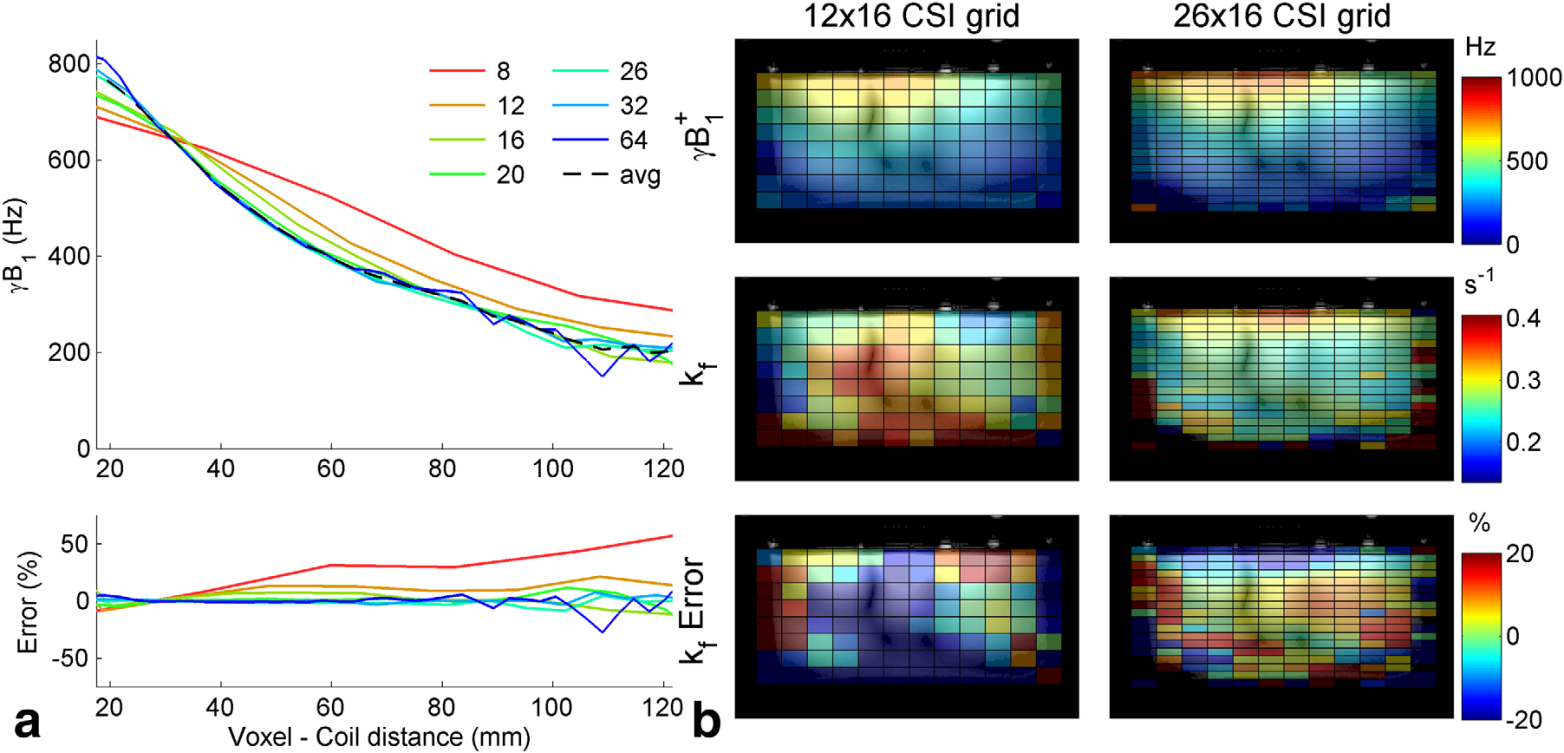
**(a)** $\gamma B_{1}^{+}$ profile for the central column of the chemical shift imaging matrix for each resolution measured. The average $B_{1}^{+}$, calculated from the resolutions ≥ 20 × 16 × 16, is shown as a black dashed line. The error relative to the average is shown in the bottom plot. **(b)** Color maps of the $B_{1}^{+}$ maps, simulated $k_{f}^{\text{CK}}$ maps, and the error in the simulated $k_{f}^{\text{CK}}$.

Compared to the true $B_{1}^{+}$ (the average of the 20, 26, 32, and 64 step maps), less than a 10% error in $B_{1}^{+}$ was observed for resolutions ≥16 at all distances from the coil (Fig. 4a). Less than 1% error was observed for resolutions ≥26 at distances less than 60 mm from the coil elements; at greater distances, errors were dominated by random errors in the Bloch‐Siegert $B_{1}^{+}$ maps due to low SNR.

In the simulation of the BOAST measurement (Fig. 4b), 78% of voxels within the phantom volume had an absolute $k_{f}^{\text{CK}}$ error greater than 20% when the CSI matrix had a resolution of 12 × 16 × 16. This dropped to 55% of voxels for the 16 × 16 × 16; voxels with high error tended to be located around the edges of the phantom. The resolution with the most low error voxels ( < 20% error) was 26 × 16 × 16 (60%).

In these two simulation experiments, significant errors are observed for CSI grid resolutions below 16 (nominal voxel size 15–12.5 mm A‐P). While at resolutions ≥26, the maps became SNR‐limited for 15‐min scan duration. We therefore chose 16 × 16 × 8 (FOV = 240 × 240 × 200 mm^3^) for the in vivo scans.

### 3.3 Skeletal Muscle Validation

#### 3.3.1 Volume Coil (3T)

The total number of voxels analyzed across the four central CSI slices was 207. The mean ( ± SD here and below) deviation from the target flip angle was 5.9 ± 4.2%.

**Figure 5:**
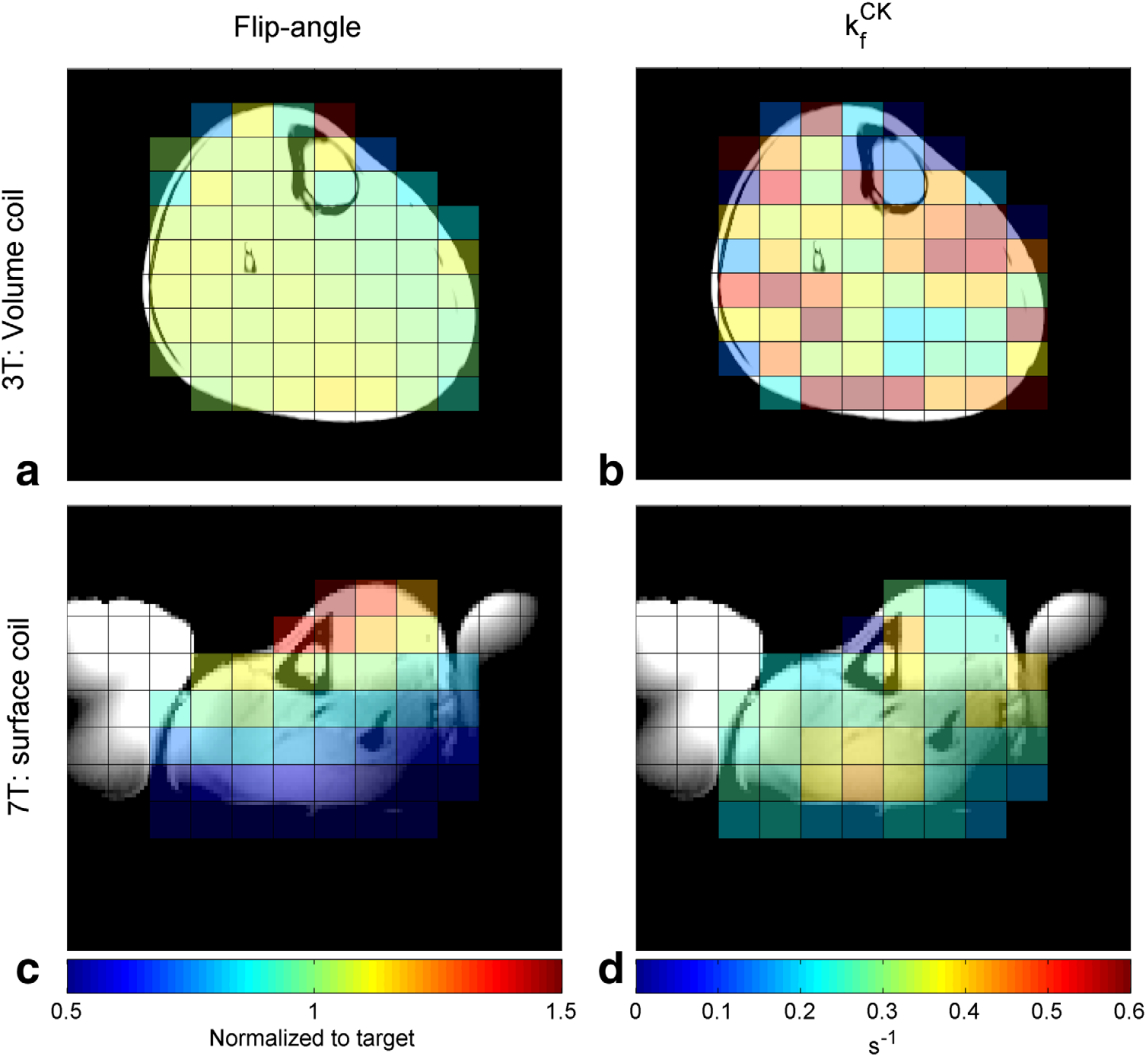
Flip‐angle **(a)** and $k_{f}^{\text{CK}}$ **(b)** maps from the central slice of a three‐dimensional chemical shift imaging matrix in healthy volunteers calves, acquired at 3T, using a volume coil. The maps are masked by the outer anatomical border. Flip‐angle map **(c)** and $k_{f}^{\text{CK}}$ map **(d)** at 7T using a surface coil. Saline bags, used for additional coil loading, are visible in the localizer images at 7T. CK, creatine kinase; T, tesla.

The CRLB^2^‐weighted mean of $k_{f}^{\text{CK}}$ was 0.31 ± 0.02 s^−1^. The mean percentage CRLB of $k_{f}^{\text{CK}}$ was 84 ± 150%, although in the central 50 voxels of the analyzed volume, the mean CRLB was 36 ± 11%. The average matched filtered SNR of PCr in the unsaturated scan (step 3, using the β flip angle) was 22 ± 12. The flip angle, normalized to the target angle, and the calculated $k_{f}^{\text{CK}}$ is shown in Figures 5a and b. The mean Q was 0.66 ± 0.20.

#### 3.3.2 Surface Coil (7T)

The total number of voxels analyzed across the five central slices was 225, with 81 voxels, within 20% of the target flip, selected for a subgroup analysis. The average deviation from the target flip angle in all voxels was 29.1 ± 17.7% and 10.1 ± 5.8% in the subgroup.

The CRLB^2^‐weighted mean of $k_{f}^{\text{CK}}$ was 0.273 ± 0.004 s^−1^ and 0.281 ± 0.002 s^−1^ in the selected voxels. For the full and masked voxel selection, the mean percentage CRLB of $k_{f}^{\text{CK}}$ was 51 ± 138% and 29 ± 81%; matched filtered SNR of PCr was 39 ± 20 and 41 ± 21; and the Q factor was 0.97 ± 0.21 and 0.98 ± 0.21. Maps from a single slice are shown in Figures 5c and d, whereas the per‐voxel results of the skeletal muscle validation can be found in the supporting information (Supporting Fig. S2).

### 3.4 Cardiac

Each of the 10 subjects produced analyzable spectra in the target voxel in each component acquisition (single‐subject example in Figs. 1c‐e). The spectra arising from the four septal voxels neighboring the target voxel were also analyzed in all 10 subjects. In the target voxel, the mean PCr SNR in the acquisitions using the β‐flip angle, with control saturation, was 13 ± 3.4. The median Q in the target voxels was 0.95, with an interquartile range of 0.13.

**Figure 6:**
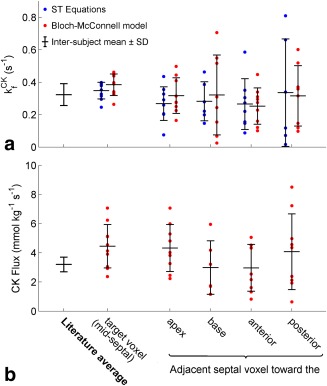
**(a)** Measured cardiac $k_{f}^{\text{CK}}$ values in the target voxel and nearest neighbor septal voxels of the 10 volunteers. Each dot is the value in the voxel, as calculated by Equation [4] (blue) and the least‐squares fitted Bloch‐McConnell model (red). The black bars give unweighted intersubject means and SDs. The mean and average SD of published literature values (Table 1). **(b)** Cardiac CK flux values in the target and nearest neighbor voxel. CK flux was calculated using Equation [5] and the literature values of [ATP] in Table 2. ATP, adenosine triphosphate; CK, creatine kinase; SD, standard deviation; ST, saturation transfer.

Calculation of $k_{f}^{\text{CK}}$ using the saturation transfer equations yielded an intersubject, CRLB^2^‐weighted, mean $k_{f}^{\text{CK}}$ of 0.35 ± 0.05 s^−1^; and least‐squares fitting to the Bloch equations gave $k_{f}^{\text{CK}}$ of 0.37 ± 0.04 s^−1^. The individual subject values are plotted for both methods in Figure 6a.

Using the results of the least squares fitting, including the PCr/ATP ratio and the average literature value of [ATP], the PCr concentration and CK flux were calculated. The per‐subject values are given in Table 3 and Figure 6b. The study average PCr concentration was 11.64 ± 2.65 mmol kg^−1^ of wet weight, and the CK flux was 4.55 ± 1.52 mmol kg^−1^ s^−1^.

**Table 3:** Results of the Least‐Squares Fitting of the Cardiac Data.

Subject	PCr/ATP	[PCr] (mmol kg^−1^ wwt)	$k_{f}^{\text{CK}}$ (s^−1^)	CK Flux (mmol kg^−1^ s^−1^)
1	1.78 ± 0.33	10.39 ± 1.94	0.40 ± 0.20	4.13 ± 2.18
2	1.50 ± 0.25	8.72 ± 1.48	0.34 ± 0.11	2.93 ± 1.06
3	1.49 ± 0.18	8.70 ± 1.04	0.45 ± 0.16	3.91 ± 1.50
4	2.25 ± 0.18	13.10 ± 1.03	0.39 ± 0.08	5.11 ± 1.07
5	1.91 ± 0.35	11.10 ± 2.03	0.46 ± 0.27	5.13 ± 3.16
6	1.61 ± 0.29	9.37 ± 1.68	0.33 ± 0.14	3.05 ± 1.39
7	1.55 ± 0.24	9.03 ± 1.42	0.26 ± 0.14	2.38 ± 1.31
8	2.29 ± 1.27	13.33 ± 7.42	0.34 ± 0.24	4.55 ± 4.11
9	2.45 ± 1.05	14.23 ± 6.13	0.44 ± 0.99	6.24 ± 14.29
10	2.74 ± 0.36	15.92 ± 2.07	0.44 ± 0.14	7.07 ± 2.40
Intersubject mean ± SD	1.96 ± 0.44	11.39 ± 2.59	0.38 ± 0.07	4.45 ± 1.49

ATP, adenosine triphosphate; CK, creatine kinase; PCr, phosphocreatine; SD = standard deviation.

## 4 DISCUSSION

The BOAST method enables measurement of the cardiac CK rate constants at 7T, and with 3D localization, for the first time. The method overcomes the current inability of surface coils to produce a B_1_‐insensitive adiabatic pulse in the myocardium at 7T. Instead, by combining Bloch‐Siegert $B_{1}^{+}$ mapping with amplitude‐modulated excitation pulses, a known (but spatially varying) flip angle is used. The BOAST method achieves a comparable precision to FAST and TRiST at 3T (both with 1D localization), with a much greater spatial resolution, in a scan time acceptable for research in volunteers and patients with mild‐to‐moderate heart disease, that is, in less than 90 min. 82 minutes is comparable to the time taken to perform TRiST (84 ± 10 min) 13, which has been successfully applied in heart failure patients.

The $k_{f}^{\text{CK}}$ measured in the cardiac experiments closely matched literature values (Table 1). The values in this study have been obtained from voxels centered on myocardium in the midinterventricular septum, with voxel sizes, calculated as the FWHM of the point‐spread‐function and at the 50% sensitivity limit of the transmit loop coil—less than half those obtained in any previous study (110 mL vs. 298 mL in 12, using the known coil geometry 30). The 3D‐CSI sequence used here has a 5.6 mL nominal voxel volume, with a well‐defined point‐spread‐function and shape that is not dependent on the receive coil geometry.

Monte Carlo Bloch simulations show that correctly choosing the T_R_ and flip angle is important, particularly in low SNR conditions, because the range of flip angles that gives acceptable mean error in $k_{f}^{\text{CK}}$ is narrow. These simulations are corroborated by the results in skeletal muscle. For both 3T and 7T experiments, the average $k_{f}^{\text{CK}}$ agrees with the literature measurements that span 0.23 s^−1^ to 0.35 s^−?1^ 17, 31 (or 0.459 s^−1^ if inversion transfer is included 32). At 3T, despite the low variation in flip angle, the SNR is relatively low compared to 7T and a correspondingly high estimated CRLB, and high intervoxel variance of $k_{f}^{\text{CK}}$ is observed. At 7T, the very high SNR results in a relatively stable $k_{f}^{\text{CK}}$ measurement despite the large spatial variation in flip angles (Sup. Fig. S3). The cardiac experiment has intermediate SNR, resulting in stable measurements in the target voxel but increasingly high variance measurements in voxels further from the target. Nevertheless, these results confirm that the BOAST method is viable for in vivo measurement.

Parameter optimization indicated that short repetition times minimize $k_{f}^{\text{CK}}$ mean error, but this must be balanced with the need for a sufficiently high‐resolution spectrum, governed by the readout duration, and a sufficiently high ratio of saturation time to readout time to achieve a saturated steady state. In the 7T acquisitions presented here, complete saturation of γ‐ATP ( $S_{\text{sat}}/S_{0}<0.1$) was achieved simultaneously with a high Q. The optimization step might be improved in future by separately optimizing the flip angles for each of the two dual‐angle T_1_ measurements.

Least squares fitting of the data reduced the uncertainty in the measured $k_{f}^{\text{CK}}$ and also enabled the calculation of PCr/ATP, and subsequent derived parameters, without introducing a systematic error associated with saturation correction of the exchanging spectra 28.

B_0_ shimming was not carried out in any of the experiments in this work due to the difficulty of obtaining adequate ^1^H images of the heart at 7T and the separation of ^1^H and ^31^P coils. With more sophisticated coils, B_0_ shimming is feasible, and we anticipate that it would improve BOAST data quality 33.

The FAST method has been deployed in a number of studies (Table 1) in patients with heart disease 7, 8, 34; $k_{f}^{\text{CK}}$ values have been shown to be a more sensitive indicator of heart failure than the PCr/ATP ratio 8, 35. Furthermore, CK flux values have been shown to be even more sensitive than $k_{f}^{\text{CK}}$ values 7, 8, 34, 35. The BOAST method will open up the possibility of measurements of regional myocardial $k_{f}^{\text{CK}}$ values, making the technique more relevant to regional myocardial disease.

### 4.1 Limitations

#### 4.1.1 $B_{1}^{+}$ Error

**Figure 7:**
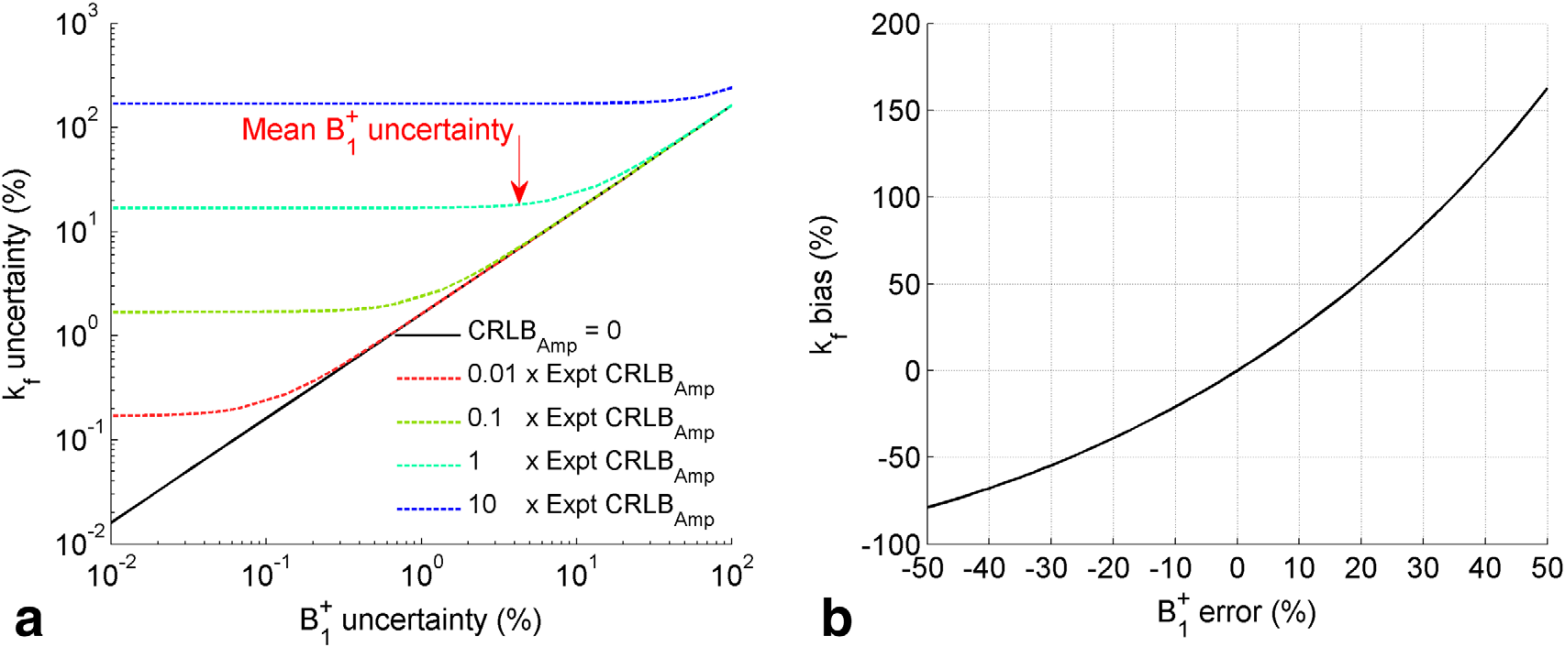
(a) $k_{f}^{\text{CK}}$ precision as a function of uncertainty in the Bloch‐Siegert $B_{1}^{+}$ measurement precision in the presence of a range of different four‐angle saturation transfer acquisition signal‐to‐noise ratio levels. (b) Error introduced to the $k_{f}^{\text{CK}}$ measurement due to under or overestimation of the $B_{1}^{+}$. CK, creatine kinase. Expt CRLB_Amp, experimental amplitude Cramér—Rao lower bound.

Propagation of error analysis of Equations [(1), (2), (3), (4)] indicated that the uncertainty in the $k_{f}^{\text{CK}}$ measurement was balanced equally between the uncertainty in the measured $B_{1}^{+}$ (4.3 ± 2.5% [mean ± SD]) and the uncertainty of the PCr amplitudes in steps 6 to 9 (2.5%–5.9%) (Fig. 7a). The analysis indicated that our choice of the relative scan time for Bloch‐Siegert B_1_‐mapping and the later FAST acquisitions was appropriate.

The simulations at different resolutions demonstrated that the method is sensitive to the effects of the PSF. Although the PSF is symmetrical around the voxel center, the RF coil transmit and receive fields of the 10‐cm loop surface coil are not. Therefore, the voxel size will affect the Bloch‐Siegert measured $B_{1}^{+}$ and eventual $k_{f}^{\text{CK}}$ measurement. This is important because a small bias in the measured $B_{1}^{+}$ results in much larger errors in $k_{f}^{\text{CK}}$ (Fig. 7b). Minimizing voxels sizes also reduces the effect of Bloch‐Siegert sensitization on intravoxel dephasing, which with surface coils can result in differing signal magnitudes for the two Bloch‐Siegert acquisitions. This imposes a limit on the size of a nominal voxel, although small voxels sizes result in a higher number of averages required for acceptable SNR and thus necessitate longer scans.

Regardless of the necessity for 3D localization (3D‐CSI, or slice‐selected 2D‐CSI) with Bloch‐Siegert $B_{1}^{+}$ mapping, it is also desirable to reduce spectral contamination from nonmyocardial tissue, for example, blood, skeletal muscle, and liver. Additionally the reduced voxel size of 3D‐CSI compared to 1D‐CSI will tend to reduce $B_{0}$ inhomogeneity across the voxel, resulting in a reduction of metabolite linewidth and fitting uncertainty.

#### 4.1.2 Surface Coil $B_{1}^{+}$ Inhomogeneity

The use of B_1_‐sensitive amplitude modulated pulses in BOAST results in a strong $B_{1}^{+}$ dependence on measurement accuracy and precision. The range of $B_{1}^{+}$ values created by the 10‐cm loop surface coil in the leg spans a large range, from approximately 50% to 150% of the target value (Fig. 5c). The low SNR in the heart (which decreases with increasing distance from the coil), the smaller and less homogenous target anatomy, and the low homogeneity of the excitation profile limited the number of voxels appropriate for analysis. Although achieving the desired 3D localization, this study only achieved sensitivity around the target voxel. We believe that the target region could be shifted to any chosen myocardial segment. However, in this study the institutional restrictions on total scan time limited us to select only a single target voxel per subject, which we chose to position in the interventricular septum following normal practice.

The situation could be improved by a change in hardware. A dedicated receive coil with higher SNR, such as a 16‐channel flexible array 21, would result in both higher measurement precision and greater tolerated range of flip angles. More uniform excitation would also reduce the range of flip angles experienced across the heart, increasing the number of voxels with acceptable sensitivity and moving the method toward whole heart sensitivity in a single scan. Volume excitation would greatly reduce the inhomogeneity; typical 3T body coils achieve flip‐angle variation between 20% to 50% across the myocardium and allow use of a dedicated receive coil 36. Whole body volume ^31^P excitation has recently been demonstrated 37.

#### 4.1.3 Blood and NADH Correction

In this work, the effects of ATP signal originating in the blood and nicotinamide adenine dinucleotide (NADH) signals that occur in close proximity to α‐ATP have been ignored. Whereas the calculation of $k_{f}^{\text{CK}}$ using the saturation transfer equations would not be affected by these factors, the least‐squares method relies upon the ATP amplitudes. The ATP peaks include a contribution from the blood of approximately 11% of the saturation‐corrected 2,3‐DPG signal, and the α‐ATP peak contains up to 20% contribution from the NADH peak^38^. Although correction is theoretically possible, both the flowing nature of blood and the lack of known T_1_ values for 2,3‐DPG and NADH mean that the unsaturated peak areas currently cannot be calculated. Thus, correction for both the partially saturated 2,3‐DPG peak amplitudes and NADH is difficult and has not been attempted.

#### 4.1.4 PCr Concentration

Currently, the PCr concentration and the CK flux are calculated using the PCr/ATP ratio and a literature value for ATP concentration. The average PCr concentration measured by this method, 11.39 ± 2.59 mmol kg^−1^, is higher than the average literature value of 9.6 ± 0.4 mmol kg^−1^. Using this average literature, PCr concentration would give an average CK flux of 3.69 ± 0.63 mmol kg^−1^ s^−1^. This is closer but still different than the literature flux of 3.2 ± 0.5 mmol kg^−1^ s^−1^.

## 5 CONCLUSION

The FAST method has been successfully adapted to allow the first three‐dimensionally localized CK forward rate constant measurements in the human heart. Values of $k_{f}^{\text{CK}}$ were measured in 10 volunteers, in less than 90 min, at 7T. The values (0.35 ± 0.05 s^−1^) agreed closely with previously measured values at lower field strengths (literature mean: 0.33 ± 0.02 s^−1^).

Although 7T ^31^P‐MRS brings significant SNR benefits, tighter constraints on $B_{1}^{+}$ presently limit the range of possible excitation pulses. This study has shown that the strategy of replacing B_1_‐insensitive pulses with amplitude‐modulated pulses calibrated by in vivo Bloch‐Siegert B_1_‐mapping is viable for human cardiac studies at 7T.

## Supporting information

Additional supporting information may be found in the online version of this article.

**Table S1.** Survey of possible methods for and CK flux measurement at 7T with 3D‐CSI localization. The localization scheme and total time for each method is taken from the referenced work. Compatibility section is colour coded to mark incompatibility (red), compatible only with modification (orange) and compatibility (green). References in the second column are to papers in the main manuscript's References section.”
**Fig. S2.** Per voxel measured $k_{f}^{\text{CK}}$ (a) and CK Flux (b) values from the two single‐volunteer skeletal muscle validations i.e. at 3T and 7T. Each dot denotes an individual voxel measurement, the black bars are the unweighted inter‐voxel mean and standard deviations. The red bars are a CRLB^2^ weighted average of the values. The results are compared with a mean and standard deviation of literature values in skeletal muscle.“
**Fig. S3.** Example spectra (a&c) from the 3T and 7T skeletal muscle validation. The spectra are taken from a centrally located voxel containing only muscle tissue in the localizer images. Also shown (b&d) are estimates of the accuracy (bias) and precision (SD) as a function of the α flip‐angle (β = 4α) for experiments with the same SNR as shown in (a&c). In panels b&d the range (mean±SD) of flip‐angles actually experienced in the skeletal muscle are marked.“

## Appendix A Least‐squares fitting

### A.1

As well as using the closed‐form Equations [(1), (2), (3), (4)] to calculate $k_{f}^{\text{CK}}$ the parameters $M_{0}^{\text{PCr}},M_{0}^{\text{ATP}},T_{1}^{*,PCr},T_{1}^{*,\gamma ‐\text{ATP}},T_{1}^{\alpha ‐\text{ATP}}$ and $k_{f}^{\text{CK}}$ were obtained by least‐squares fitting of the PCr, unsaturated γ‐ATP, and α‐ATP peaks by the following equations:

(A.1) $$M^{\text{PCr}}\left(T_{R},\theta \right)=M_{0}^{\text{PCr}}\;\sin\;\theta \left(1+q_{1}\frac{\left(1-\cos\;\theta \right)\;\exp\;\left(D_{+}T_{R}\right)}{\left(1-\cos\;\theta \right)\;\exp\;\left(D_{+}T_{R}\right))}+q_{2}\frac{\left(1-\cos\;\theta \right)\;\exp\;\left(D_{-}T_{R}\right)}{\left(1-\cos\;\theta \right)\;\exp\;\left(D_{-}T_{R}\right))}\right),$$

(A.2) $$M^{\gamma ‐\text{ATP}}\left(T_{R},\theta \right)=M_{0}^{\gamma ‐\text{ATP}}\;\sin\;\theta \left(1+r_{1}\frac{\left(1-\cos\theta \right)\exp\left(D_{+}T_{R}\right)}{\left(1-\cos\theta \right)\exp\left(D_{+}T_{R}\right))}+r_{2}\frac{\left(1-\cos\theta \right)\exp\left(D_{-}T_{R}\right)}{\left(1-\cos\theta \right)\exp\left(D_{-}T_{R}\right))}\right),$$

where,

(A.3) $$D^{+}\left(T_{1}^{*\text{PCr}},T_{1}^{*\gamma ‐\text{ATP}},k_{f},k_{r}\right)=0.5\left[-\left(\frac{1}{T_{1}^{*\text{PCr}}}+\frac{1}{T_{1}^{*\gamma -\text{ATP}}}+k_{f}+k_{r}\right)+disc\right],$$

(A.4) $$D^{-}=D^{+}-disc,$$

(A.5) $$disc=\sqrt{{\left(\frac{1}{T_{1}^{*\text{PCr}}}-\frac{1}{T_{1}^{*\gamma -\text{ATP}}}+k_{f}-k_{r}\right)}^{2}+4k_{f}k_{r},}$$

(A.6) $$q_{1}=0.5\left[\left(\frac{1}{T_{1}^{*\text{PCr}}}-\frac{1}{T_{1}^{*\gamma -\text{ATP}}}-k_{f}-k_{r}\right)/disc-1\right],$$

(A.7) $$q_{2}=-1-q_{1},$$

(A.8) $$r_{1}=0.5\left[\left(\frac{1}{T_{1}^{*\gamma -\text{ATP}}}-\frac{1}{T_{1}^{*\text{PCr}}}-k_{f}-k_{r}\right)/disc-1\right],$$

(A.9) $$r_{2}=-1-r_{1},$$

(A.10) $$M_{\text{SS}}^{\text{PCr}}=\frac{R_{1}^{\text{PCr}}}{R_{1}^{\text{PCr}}+k_{f}}M_{0}^{\text{PCr}},$$

(A.11) $$\frac{1}{T_{1}^{\prime \text{PCr}}}=\frac{1}{T_{1}^{*\text{PCr}}}+\frac{1}{\tau }=\frac{1}{T_{1}^{*\text{PCr}}}+k_{f}.$$

In which $T_{R}$ is the repetition time, θ is the flip‐angle, $T_{1}^{*x}$ is the intrinsic T_1_ of X, $T_{1}^{\prime \text{PCr}}$ is the T_1_ of PCr during steady‐state saturation of γ‐ATP, $R_{1}^{\text{PCr}}=1/T_{1}^{*\text{PCr}}$, and $M_{SS}^{\text{PCr}}$ is the equilibrium amplitude of the PCr peak during steady‐state saturation of γ‐ATP. $k_{f}$ is the pseudo first‐order forward rate constant, and $k_{r}$ is the reverse rate constant for the reaction:

$$\text{Mg}{.\text{ADP}}^{-}+{\text{PCr}}^{2-}+H^{+}\overset{k_{f}}{\underset{k_{r}}{⇄}}\text{Mg}{.\text{ATP}}^{2-}+\text{Cr}.$$

Equations [A.1] to [A.9] were derived by Spencer *et al*. 39
