# Supplementary material for: Creatine kinase rate constant in the human heart measured with 3D‐localization at 7 tesla
Source: Magn Reson Med. 2016 Aug 31;78(1):20–32. doi: 10.1002/mrm.26357 (PMC5484353; doi:10.1002/mrm.26357)
Supplement: Supplementary file 1 — Table S1. Survey of possible methods for and CK flux measurement at 7T with 3D‐CSI localization. The localization scheme and total time for each method is taken from the referenced work. Compatibility section is colour coded to mark incompatibility (red), compatible only with modification (orange) and compatibility (green). References in the second column are to papers in the main manuscript's References section.” Fig. S2. Per voxel measured kfCK (a) and CK Flux (b) values from the two single‐volunteer skeletal muscle validations i.e. at 3T and 7T. Each dot denotes an individual voxel measurement, the black bars are the unweighted inter‐voxel mean and standard deviations. The red bars are a CRLB2 weighted average of the values. The results are compared with a mean and standard deviation of literature values in skeletal muscle.“ Fig. S3. Example spectra (a&c) from the 3T and 7T skeletal muscle validation. The spectra are taken from a centrally located voxel containing only muscle tissue in the localizer images. Also shown (b&d) are estimates of the accuracy (bias) and precision (SD) as a function of the α flip‐angle (β = 4α) for experiments with the same SNR as shown in (a&c). In panels b&d the range (mean±SD) of flip‐angles actually experienced in the skeletal muscle are marked.“ [file MRM-78-20-s001.docx]

| **Method** | **Reference** | **Method description** | **Localisation** | **Total time**  **(min)** | **Compatible with:** | |
| --- | --- | --- | --- | --- | --- | --- |
|  |  |  |  |  | **3D localisation?** | **7T RF limitations?** |
| A | (14) | **Time dependent saturation transfer (TDST)**   1. Progressive saturation: Multiple long T_R_ (12 s) acquisitions to determine.    1. Long T_R_ (12 s) determination of $M_{\mathrm{ss}}$ and $M_{\mathrm{Ctrl}}$. | 1D-ISIS | 60 | No – long T_R_ is incompatible with 3D-CSI. T_A_ > 1 hour per step. | Requires flip angle calibration or 90° B_1_-insensitive pulses. |
| B | (17) | **Steady-state saturation transfer**   1. Inversion recovery + selective saturation to measure $T_{1}'$ . 2. Long T_R_ determination of $M_{\mathrm{ss}}$ and $M_{\mathrm{Ctrl}}$. 3. Determine $k_{f}$ from Eq. [4]. | None | 10.5 | No – long T_R_ is incompatible with 3D-CSI. T_A_ > 1 hour per step. See F,G and H. | Requires flip angle calibration or 90° B_1_-insensitive pulses. |
| C | (16) | **Inversion transfer**   1. Single inversion recovery experiment monitoring magnetisation of exchanging peaks. 2. Multi-parametric fit to Bloch-McConnell equations. | None | 36 | Possibly - Inversion recovery incompatible. Look-Locker compatible with 3D CSI (requires FA calibration).  Low number of acquisitions. | Inversion possible with hyper-secant, WURST or GOIA pulses. Look-Locker would require flip-angle calibration. |
| D | (18) | **Optimised approach to steady state**   1. Long T_R_ determination of $M_{0}$. 2. Measure $M_{\mathrm{ss}}$ directly with short T_R_ (5.5 s) via optimised approach to steady-state equilibrium, having previously determined $T_{1}^{*}$. 3. Short T_R_ scan + step 2. to measure $T_{1}'$.(46) 4. Determine $k_{f}$ from Eq. [4]. | None | 4.3 | Long T_R_ steps are compatible with 1D-CSI (see G & H), but are incompatible with 3D-CSI for which a single step takes longer than 1 hour. | Requires knowledge of exchange parameters i.e. intrinsic T_1_ and metabolite pool size, which are not known for myocardial tissue at 7T. |
| E | (19) | **Iterative** $\boldsymbol{T}_{\mathbf{1}}^{\mathbf{nom}}$ **method**   1. Determine $T_{1}^{\mathrm{nom}}$ – T_1_ under selective and partial saturation, with SNR optimised flip angle and T_R_, from pool size and $T_{1}^{*}$ estimate. 2. Measure $M'$ and $M$ at same flip angle and T_R_. 3. Iteratively fit to $\frac{M}{M^{'}}=\beta+T_{1}^{\mathrm{nom}}k_{f}$, updating estimate of $T_{1}^{\mathrm{nom}}$. | 1D-CSI | 13.6 | Compatible with 3D-CSI.  Low number of acquisitions: 2. | Requires knowledge of exchange parameters i.e. intrinsic T_1,_ and metabolite pool size; which are not known for myocardial tissue at 7T. |
| F | (11) | **Four-angle saturation transfer (FAST)**   1. Dual angle ($\alpha=15^{\circ} \& 60^{\circ}, T_{R}=1 s)$ measurement of $T_{1}'$ (with selective saturation).(25) 2. Dual angle measurement of apparent $T_{1}$ (with control selective saturation). 3. Calculate $M_{\mathrm{Ctrl}}$ and $M_{\mathrm{SS}}$ from partially saturated magnetisation in 1. & 2. 4. Determine $k_{f}$ from Eq. [4]. | 1D-CSI | 39 | Compatible with 3D-CSI.  Low number of acquisitions: 4. | Requires flip-angle calibration or 15°/60° B_1_^+^-insensitive pulses. |
| G | (12) | **Triple repetition time saturation transfer (TRiST)**   1. Dual-T_R_ ($T_{R}=2 s \& 10 s,\alpha=90^{\circ}$) measurement of $T_{1}'$. 2. Long T_R_ ($T_{R}=15 s$) measurement of $M_{\mathrm{ctrl}}$. 3. Optional long T_R_ ($T_{R}=15 s$) measurement of $M_{0}$. 4. Determine $k_{f}$ from Eq. [4]. | 1D-CSI | 45 | Incompatible with 3D-CSI – T_A_ > 1 hour per step.  Low number of acquisitions: 4. | Requires flip-angle calibration or 90° B_1_-insensitive pulses. |
| H | (13) | **Two repetition time saturation transfer (TwiST)**   1. Use literature values of $T_{1}^{*}$. 2. Long T_R_ ($T_{R}=15 s$) measurement of $M_{\mathrm{ctrl}}$. 3. Long T_R_ ($T_{R}=15 s$) measurement of $M_{\mathrm{SSl}}$. 4. Optional long T_R_ ($T_{R}=15 s$) measurement of $M_{0}$. 5. Determine $k_{f}$ from modified Eq [4]. | 1D-CSI | 30 | Incompatible with 3D-CSI – T_A_ > 1 hour per step.  Low number of acquisitions: 3. | Requires flip-angle calibration or 90° B_1_-insensitive pulses.  Requires knowledge of intrinsic PCr T_1_, which has not been measured at 7T. |

Supporting Table S1 Survey of possible methods for and CK flux measurement at 7T with 3D-CSI localisation. The localisation scheme and total time for each method is taken from the referenced work. Compatibility section is colour coded to mark incompatibility (red), compatible only with modification (orange) and compatibility (green). References in the second column are to papers in the main manuscript’s References section.

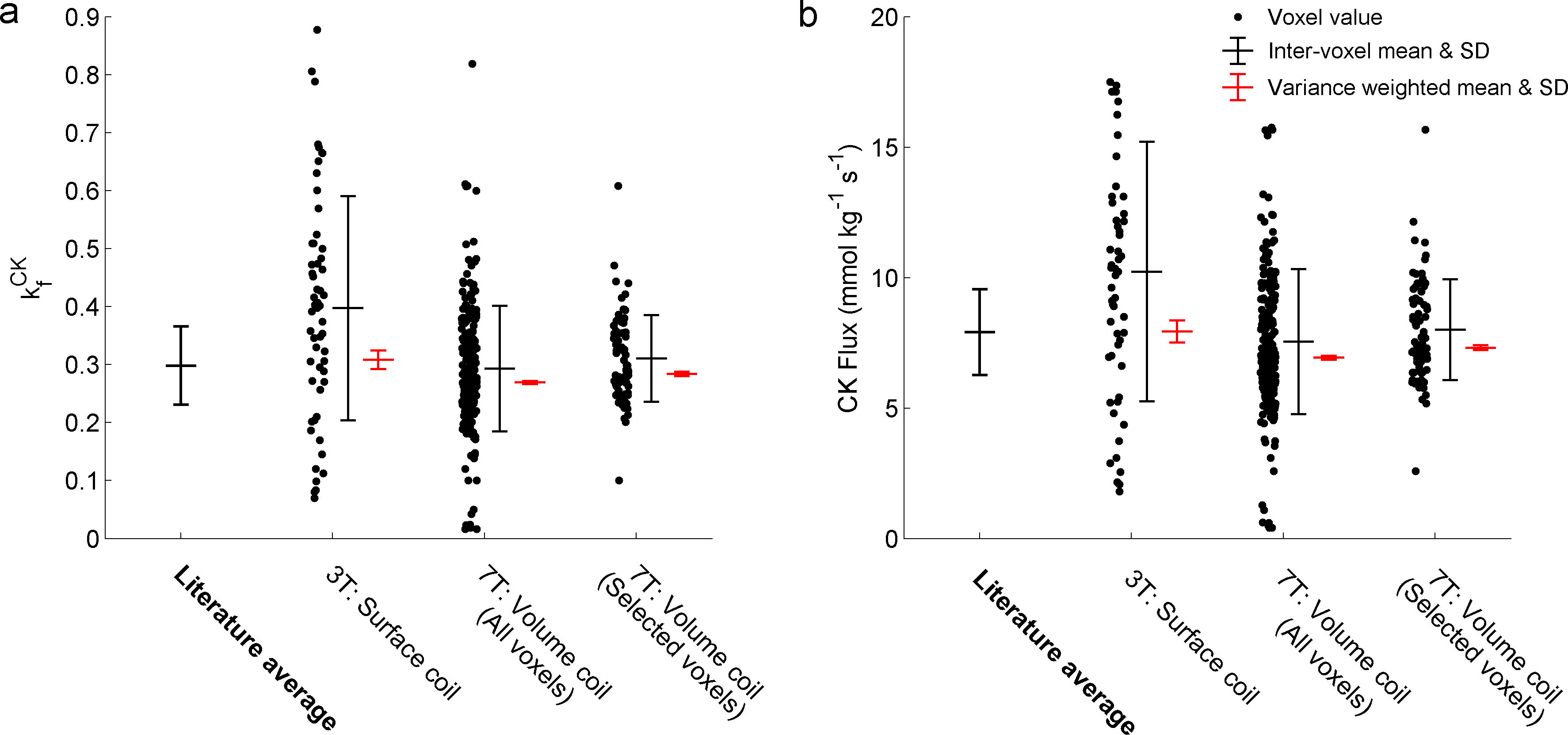


Supporting Figure S2 Per voxel measured $k_{f}^{\mathrm{CK}}$ (**a**) and CK Flux (**b**) values from the two single-volunteer skeletal muscle validations i.e. at 3T and 7T. Each dot denotes an individual voxel measurement, the black bars are the unweighted inter-voxel mean and standard deviations. The red bars are a CRLB^2^ weighted average of the values. The results are compared with a mean and standard deviation of literature values in skeletal muscle.


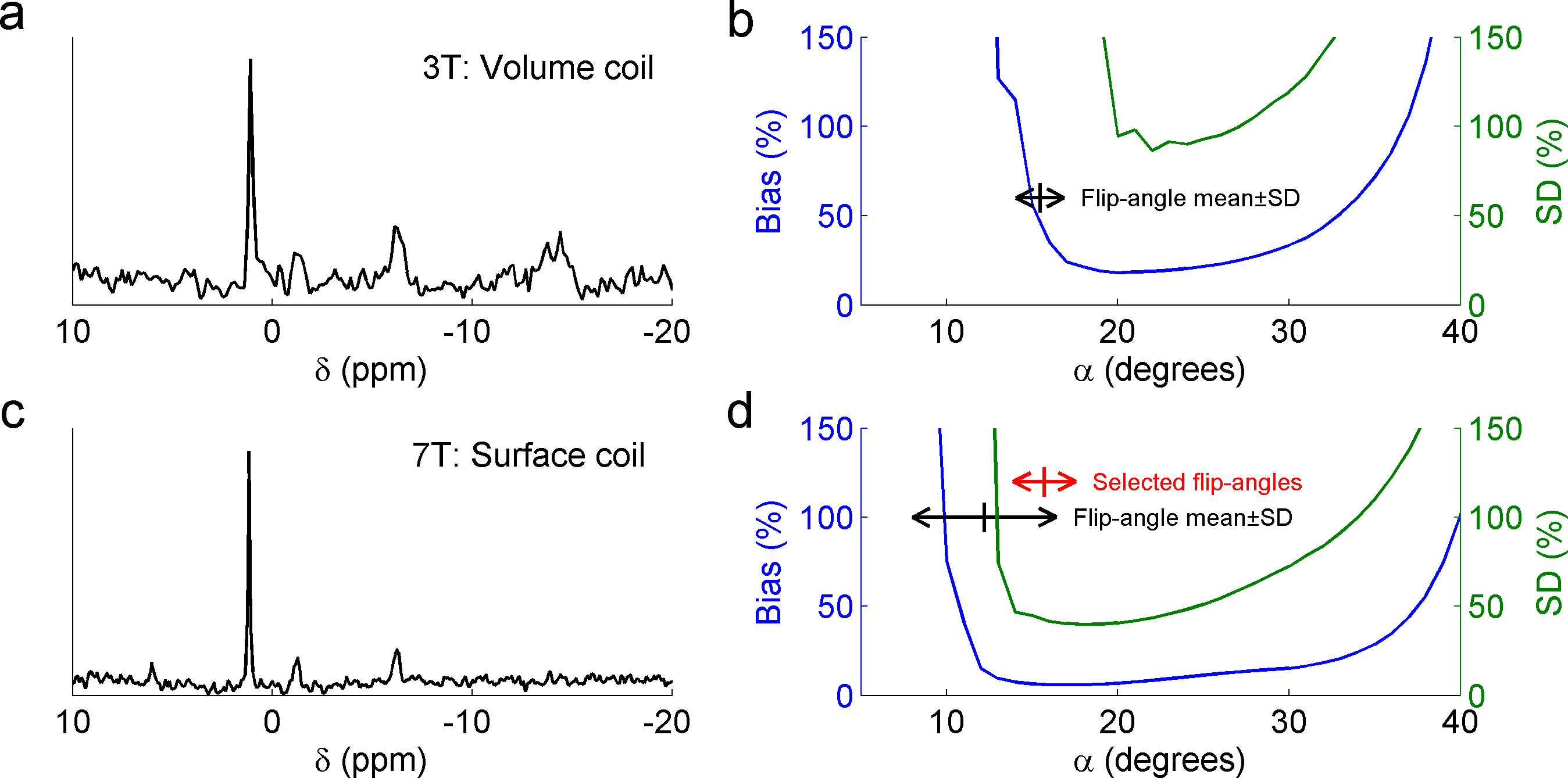


Supporting Figure S3 Example spectra (**a&c**) from the 3T and 7T skeletal muscle validation. The spectra are taken from a centrally located voxel containing only muscle tissue in the localiser images. Also shown (**b&d**) are estimates of the accuracy (bias) and precision (SD) as a function of the α flip-angle (β = 4α) for experiments with the same SNR as shown in (**a&c**). In panels **b&d** the range (mean±SD) of flip-angles actually experienced in the skeletal muscle are marked.
